# WebCircRNA: Classifying the Circular RNA Potential of Coding and Noncoding RNA

**DOI:** 10.3390/genes9110536

**Published:** 2018-11-06

**Authors:** Xiaoyong Pan, Kai Xiong, Christian Anthon, Poul Hyttel, Kristine K. Freude, Lars Juhl Jensen, Jan Gorodkin

**Affiliations:** 1 Center for Non-Coding RNA in Technology and Health, University of Copenhagen, 1870 Frederiksberg C, Denmark; xypan172436@gmail.com (X.P.); anthon@rth.dk (C.A.); 2 Department of Veterinary and Animal Sciences, University of Copenhagen, 1870 Frederiksberg C, Denmark; hpw927@alumni.ku.dk (K.X.); poh@sund.ku.dk (P.H.); kkf@sund.ku.dk (K.K.F.); 3 Department of Disease Systems Biology, Novo Nordisk Foundation Center for Protein Research, University of Copenhagen, 2200 Copenhagen, Denmark; 4 BrainStem—Stem Cell Center of Excellence in Neurology, University of Copenhagen, 1870 Frederiksberg C, Denmark

**Keywords:** circular RNA, random forest, noncoding RNA

## Abstract

Circular RNAs (circRNAs) are increasingly recognized to play crucial roles in post-transcriptional gene regulation including functioning as microRNA (miRNA) sponges or as wide-spread regulators, for example in stem cell differentiation. It is therefore highly relevant to identify if a transcript of interest can also function as a circRNA. Here, we present a user-friendly web server that predicts if coding and noncoding RNAs have circRNA isoforms and whether circRNAs are expressed in stem cells. The predictions are made by random forest models using sequence-derived features as input. The output scores are converted to fractiles, which are used to assess the circRNA and stem cell potential. The performances of the three models are reported as the area under the receiver operating characteristic (ROC) curve and are 0.82 for coding genes, 0.89 for long noncoding RNAs (lncRNAs) and 0.72 for stem cell expression. We present WebCircRNA for quick evaluation of human genes and transcripts for their circRNA potential, which can be essential in several contexts.

## 1. Introduction

Circular RNAs (circRNAs) were recently discovered to be widespread, abundant, expressed across species, and implicated in several diseases. They are created by non-linear backsplicing between a splice donor and an upstream splice acceptor, and evidence is emerging for them playing functional roles as microRNA (miRNA) sponges [1,2] and in regulation of gene splicing and transcription [3]. Recently, the miR-7 sponge *CDR1as* has been found to be involved in stem cell regulation of periodontal ligament [4]. Other studies suggest that circRNAs can encode proteins [5], and 90% of the 92,375 human circRNAs in the circBase database (v0.1) [6] arise from protein-coding genes (PCGs). The number of discovered circRNAs has been rapidly increasing in recent years due to the development of new high-throughput sequencing technologies, and circBase now contains more than 90,000 circRNA transcripts [6]. In addition, circRNAs are expressed in a cell/tissue-specific manner [2]; for example, 16,017 are expressed in stem cells, and they are especially prominent during embryonic development [7].

Current computational pipelines are focused on identifying presence of backsplicing junction-spanning reads from RNA-seq data [8]. Commonly, pipelines to identify circRNAs map the RNA-seq reads into a reference genome using mappers such as TopHat [9], and then use the unmapped reads to detect the backsplicing junction spanning reads. This principle is used in circRNA detection programs such as CIRCexplorer [10] and find_circ [2]. As reported by Hansen et al. 2016 [11], these tools suffer from relatively high false positive rates, and dramatic differences are observed between the various tools. In contrast to these tools, which take RNA-seq data as input, we employ a strategy based solely on predictions from the primary sequence.

Our strategy takes outset in learning sequence-derived patterns from accumulated identified circRNAs using machine learning, and apply the trained models to filter out falsely annotated circRNAs as a post-processing step. For a given sequence, our tool outputs three scores: the first two signify the potential of the transcript being a circRNA under the assumption that it is a PCG or a long noncoding RNA (lncRNA), respectively, and the third scores how likely it is to be expressed in stem cells if it is indeed a circRNA. Underlying the three respective types of output scores are three random-forest models: (i) circular RNA potential of PCGs (CP-PCG); (ii) circular RNA potential of lncRNAs (CP-lncRNA), which is based on the work in Pan and Xiong 2015 [12]; and (iii) stem cell potential of circRNAs (SP-circRNA). We introduce calibrated scoring schemes for the three types of predictions and furthermore make the method available as a user-friendly web server, which takes one or more transcripts as input, either in the form of genome coordinates or nucleotide sequences.

## 2. Materials and Methods

**Figure 1:**
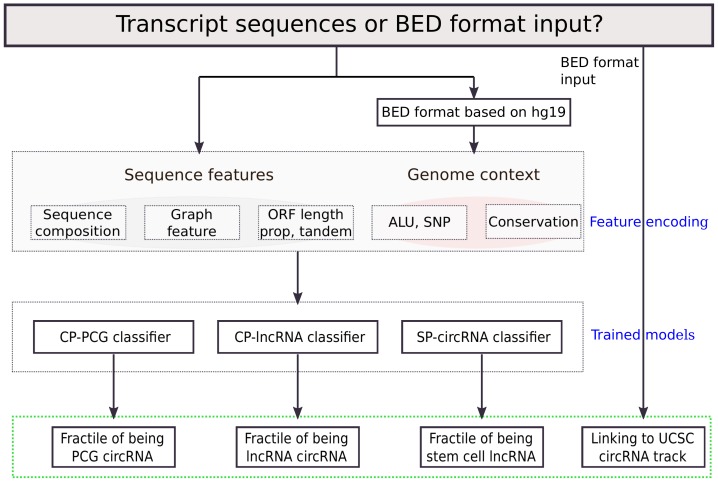
Flowchart of the WebCircRNA framework. BED: browser extensible data; ORF: open reading frame; ALU: transposable element; SNP: single nucleotide polymorphism; CP: circular RNA potential; PCG: protein coding gene; lncRNA: long non-coding RNA; SP: stem cell potential; circRNA: circular RNA; UCSC: University of California, San Diego.

In this study, we present a machine learning based method to classify the circular RNA potential for coding and non-coding RNA (Figure 1). Data from circBase and GENCODE v19 [13] were used to create the training data. From these, we extracted different features such as sequence composition and graph representations of, e.g., RNA secondary structure and conservation. We then trained random forest models to perform the classification based on the extracted features.

### 2.1. Construction of Datasets

We downloaded 92,375 circRNA transcripts from circBase [6]. For circRNAs, we removed transcripts shorter than 200 nt and overlapping circRNA transcripts, which resulted in a set of 14,084 circRNAs also used in PredcircRNA [12]. We also collected 20,345 PCGs from GENCODE v19. To ensure a clean dichotomy for training, we removed the PCGs overlapping circRNAs in circBase resulting in 9533 PCGs used in the following. We also extracted the lncRNAs using the same processing scheme as for PCGs, resulting in 19,722 lncRNAs. We randomly selected 10,000 for training lncRNAs and used the remaining 9722 lncRNAs for independent testing. Of the 10,000 training lncRNAs, 3500 are lincRNAs and 75 overlap with PCGs. Next, we constructed the dataset for circRNAs versus PCGs. We randomly selected 10,000 circRNAs and 8000 PCGs for training and used the remaining 4084 circRNA and 1533 PCGs as independent test set. For stem-cell dataset, we obtained 2082 circRNAs only expressed in H1hsec and randomly selected the same number of other circRNAs from other cell lines, not expressed in H1hsec. We used 1800 stem cell circRNAs and 1800 circRNAs not expressed in H1hsec as training dataset and the remaining as independent testing dataset. An overview of how these were used for training and testing is provided in Table 1.

To validate the WebCircRNA on non-human data, we collected the circRNA sequences of *Mus musculus* from circBase and the PCGs and lncRNAs of *Mus musculus* from GENCODE. We obtained 5657 mouse circRNAs, 3904 mouse lncRNAs and 16,763 mouse PCGs. We used CD-HIT [14] to remove all sequences that are more than 80% identical to a human sequence. We obtained 5397 mouse circRNAs, 3700 lncRNAs and 16,552 PCGs as mouse test data. The sequences of 5397 circRNAs and 3700 mouse lncRNAs are tested on CP-lncRNA model trained on human data. Similarly, the sequences of 5397 circRNAs and 16,552 mouse PCGs are evaluated on CP-PCG model.

### 2.2. Feature Encoding

We extracted 178 features as described below, which are summarized in Table 2. The values for each feature were normalized to the interval from 0 to 1.

*Basic sequence features.* These are made up of the features that are trivially derived from the sequence. They include the gene length, its GC content and the 64 trinucleotide frequencies. They also comprise the GT, AG, GTAG and AGGT frequencies; the rationale behind this is that the GT/AG signal associates with exon-junction [15] and backsplicing.

*Graph features.* For each sequence, we generated a set of RNA structure features using GraphProt 1.0.1 [16]. The rationale for this is that RNA structure plays key roles in gene splicing [17], which has an impact on forming circRNAs, such as backsplicing [18]. The RNA graph uses nodes to represent the nucleotides and edges to represent the bond relationships between the nucleotides. The input for GraphProt is RNA sequences, whose corresponding graphs are created using the script fasta2shrep_gspan.pl (with parameters -seq-graph-t -nostr -stdout -fasta). The resulting graph is used as input to GraphProt (using -a FEATURE) to create a set of more than 30,000 graph features. To reduce the high-dimensional graph features, we applied random forest, implemented in Scikit-learn [19], to rank importance score for graph features based on randomly selected circRNA and non-circRNA subset. By ranking the graph features based on the random forest feature score, measured on 1747 circRNAs and 1747 other RNAs, we chose the Top 101 graph features with high importance score (see Section 2.3). We tried Top 50, 101 and 200 graph features. When combining with 77 other features in Table 2, the Top 50, 101, and 200 yield similar five-fold cross-validation performance on circRNA vs. PCG training data with the area under receiver operating characteristic (AUC) values of 0.798, 0.797 and 0.793, respectively. Hence, we used the Top 101 graph features for the three models.

*Conservation features.* These were calculated from per-base phyloP conservation score [20]. For each sequence, we calculated the mean and standard deviation of the conservation scores across the length of the sequence.

*Other features.* ALU repeats can make the splice sites recognize each other, which promote circularization. Therefore, we derived the ALU frequency for each sequence from RepeatMasker track of the UCSC Genome Browser [21]. Similarly, tandem duplications within a gene can promote backsplicing; we detected them using Tandem Repeats Finder [22], and calculated the frequency of tandem repeats over the sequence. To evaluate the protein-coding potential of each sequence, we extracted the longest Open Reading Frame (ORF) using txCdsPredict from the UCSC Browser. From this, we calculated the ORF length and the ORF propensity (ORF prop), which is ORF length divided by the sequence length. Finally, SNP information is also integrated by calculating the SNP density using data from the 1000 Genomes Project [23].

### 2.3. Random Forest Models

Random forests [24] are constructed from multiple unpruned decision trees, with each tree grown from bootstrap sampling of the training data and using a random subset of the input features. During bootstrap sampling, 2/3 of data were used for decision tree training, and 1/3 of data (out-of-bag data) were used for inner validation. In this study, we used the random forest implementation from Scikit-learn [19]. We optimized the number of trees in the random forest based on the cross-validated performance. We used the following number of trees: 80 for CP-PCG, 100 for CP-lncRNA and 60 for SP-circRNA. The optimal parameters are searched in the range from 10 to 100 with a step size of 10, and we selected the value with the highest performance of a five-fold cross-validation on the training set. It should be noted that feature selection was not involved in the model optimization, as we only ranked graph features in a separate data subset, as also mentioned in Section 2.2.

Random forest models can also be used to rank the input features based on their individual contributions. During the training process, the out-of-bag (OOB) error was kept and averaged over all trees. Then, for one feature at a time, its feature values were randomly shuffled over the OOB samples, and the OOB error was re-computed. The importance score for a feature was obtained by averaging the difference in OOB error over all trees.

### 2.4. Prediction Scores

**Figure 2:**
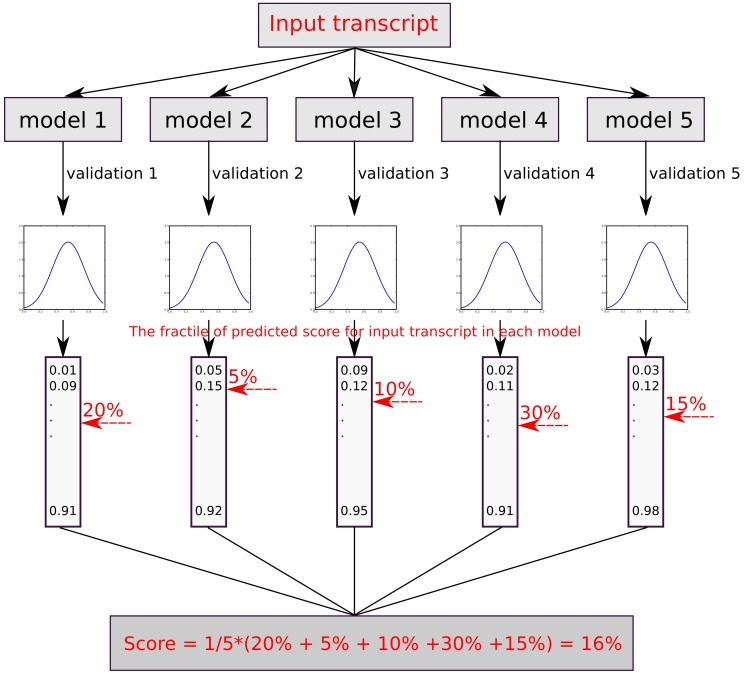
The flowchart illustrates how the final fractile score of the input sequences is obtained. Each model predicts a score which is then converted into a fractile. Novel sequences not in the validation sets are scored relative to the fractile in each model and then averaged over all five models.

The training of the three respective classifiers CP-PCG, CP-lncRNA and SP-circRNA was done using a five-fold cross validation. To calculate a combined score of the five resulting cross-validated models, we first converted the score from each model to a fractile within the score distribution of the respective validation set. Next, the average of the five fractile scores was calculated (Figure 2). Calculating the average fractile score is equivalent to calculating the average rank, since the respective lists of genes used to train each classifier are equally long within a cross-validation ensemble. What we used to combine the scores to across an ensemble of classifiers is thus rank aggregation, which is an established method in the literature [25]. For CP-PCG and CP-lncRNA, the higher is the score, the more likely the sequence is to be a circRNA. Similarly, a higher fractile score implies that a circRNA is more likely to be expressed in stem cells in the case of SP-circRNA. To calibrate the scores of the three classifiers, we estimated the false positive rate (FPR) as a function of the fractile from the score distribution on the negative data (outlined in Table 1).

## 3. Results

### 3.1. Performance Evaluation

**Figure 3:**
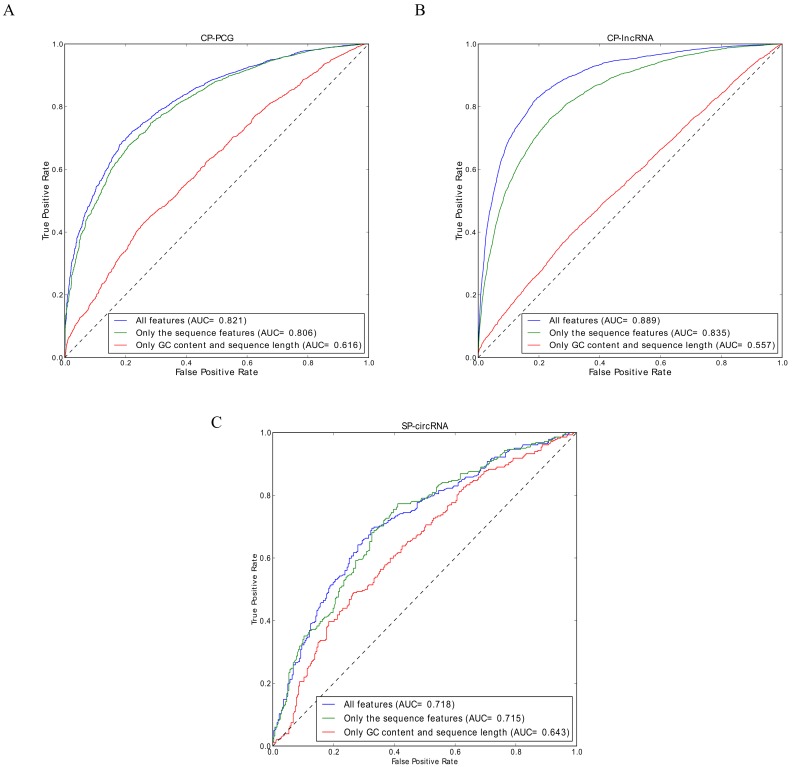
ROC curves for: (**A**) the PCG circRNA model (CP-PCG); (**B**) the lncRNA circRNA model (CP-lncRNA); and (**C**) stem cell circRNA model (SP-circRNA). In these three instances, the ROC curve using all 178 features indicated in Table 2 is compared to models using “only the GC content and sequence length” and “only the sequence features”.

To assess the quality of the predictions provided by the webcircRNA web server, we evaluated the performance of each of the three classifiers on independent test sets. Evaluating the performance of CP-PCG on the independent test set, we achieved a sensitivity of 0.736, a specificity of 0.752, and an AUC of 0.821 (Figure 3A). For CP-lncRNA, we obtained similar test set performances with a sensitivity of 0.827, a specificity of 0.806, and an AUC of 0.889 (Figure 3B). Finally, SP-circRNA presented a sensitivity of 0.688, a specificity of 0.673, and an AUC of 0.718 (Figure 3C). Here we do not compare WebCircRNA with other RNA-seq based methods (e.g., find_circ and CIRCexplore), since they take completely different starting points. WebCircRNA takes sequences of assembled transcripts as inputs, but find_circ and CIRCexplore starts from RNA-seq data. WebCircRNA can be used as a post-processing step to remove incorrectly annotated circRNAs from those RNA-seq based tools.

**Figure 4:**
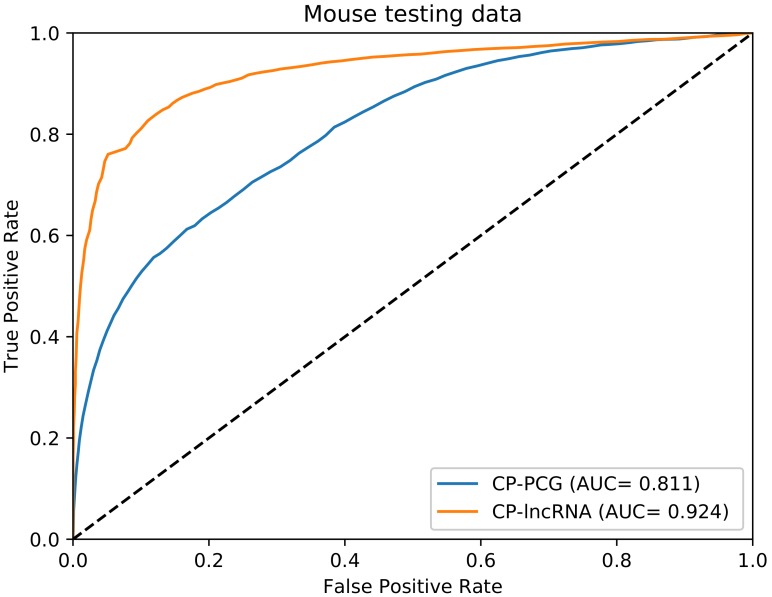
ROC curves for the testing mouse data on the CP-PCG and the CP-lncRNA, which are trained on human data using “only the sequence features”, respectively.

To test the CP-PCG and CP-lncRNA models on non-human organisms, we applied them on mouse test data. The receiver operating characteristic (ROC) figure is shown in Figure 4. We obtained performances of AUC 0.811 of CP-PCG and AUC 0.924 of CP-lncRNA, indicating the CP-PCG and CP-lncRNA models trained on human data can also be applied for other organisms.

### 3.2. Feature Importance

To test if more basic features can account for the performance, we also trained models on two simpler feature sets: one consisting only of the GC content and the sequence length, and one including all external features that we did not directly derive from the primary sequence (Table 2). The performance of these were compared to that of all features. Whereas the GC content and sequence length performs much worse than the full model (slightly better than random), leaving out the external features leads to only a minor drop in performance for CP-PCG and SP-circRNA but a notable drop for CP-lncRNA, as shown in Figure 3. These results show that it is not trivial to predict circRNAs from sequence alone. The difference in performance observed for external features between the classifiers likely reflects that PCGs are in general much more strongly conserved than lncRNAs [13], since conservation is among the external features. This highlights the importance of training separate circRNA classifiers for PCGs and lncRNAs.

**Figure 5:**
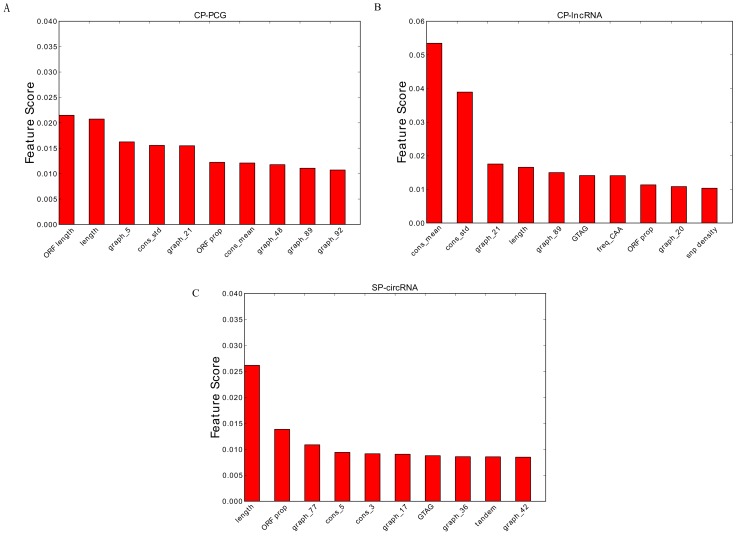
The Top 10 features for: (**A**) the CP-PCG model; (**B**) the CP-lncRNA model; and (**C**) the SP-circRNA model. Prefix graph refers to the 101 graph features, prefix cons refers to conservation feature and prefix freq refers to codon frequency feature.

This prompted us to further analyze which features are the most important for the classifiers. We therefore considered the Top-10 highest ranked features according the random forest models (Figure 5). Consistent with our performance observations, conservation features are by far the most important for lncRNAs, while they are less profound for PCGs. This is in agreement with the with observation that circRNAs often are evolutionarily conserved [26]. Conversely, sequence length matters more in the PCG and stem cell circRNA cases.

### 3.3. The WebCircRNA Web Server

Using the graphical representation of scoring CP-PCG, CP-lncRNA and SP-circRNA, the web server takes two types of input, a BED file with coordinates of the human genome (hg19) or a FASTA file. Each line in the BED file is considered as an entity on which predictions are made. Whereas this provides flexibility in terms of interfacing with genome browsers, the mode of the FASTA file allows for another form of flexibility, such as spliced sequences or sequences from organisms closely related to human. When submitting in the BED format, the sequences must be from the human genome. When submitting in FASTA mode, any sequence will be accepted, however, one should be aware that the models were trained only on human data. The WebCircRNA software can be freely downloaded on the download page of the web site.

**Figure 6:**
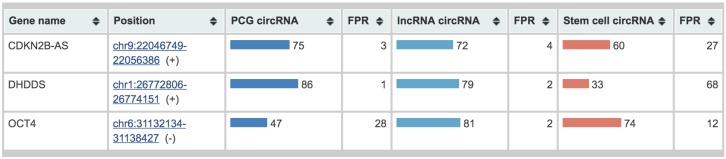
WebCircRNA. Example output for the lncRNA CDKN2B-AS and the two PCGs DHDDS and OCT4 is shown. We thus ignore the “PCG circRNA” score for CDKN2B-AS and the “lncRNAs circRNA” scores for DHDDS and OCT4. Because “stem cell circRNA” scores only apply to circRNAs, this too should be disregarded for OCT4, since it is not predicted to have a circRNA isoform. The respective “FPR” shows the estimated false positive rate of the corresponding methods. When submitting a BED file, the genomic context of any prediction can view in the UCSC browser via the link in the “Position” column.

As examples for using the web server, we present the analysis of three genes CDKN2B-AS, DHDDS and OCT4, not part of the training data. (Figure 6). CDKN2B-AS is a lncRNA gene, whose circRNA isoform is associated with atherosclerosis via changing INK4/ARF expression [27]. WebCircRNA correctly predict this as circRNA with a score of 72% as seen in the column “lncRNA circRNA”. Since we know that this gene encodes a lncRNA, the column “PCG circRNA” should be ignored. Conversely, DHDDS and OCT4 are PCGs; we thus consider the column “PCG circRNA” and not “lncRNAs circRNA”. The score of 86% for DHDDS is in agreement with it having a circRNA isoform [28]. For OCT4, we obtain a score 47%, indicating that it is unlikely to produce a circRNA isoform. To our knowledge, there is indeed currently no evidence supporting that OCT4 can be a circRNA. The “stem cell circRNA” predictions suggest that CDKN2B-AS is likely expressed in stem cells, whereas DHDDS is not. Since OCT4 is not predicted to have a circRNA isoform, the stem cell prediction is not applicable to this gene. 

## 4. Concluding Remarks

In this study, we addressed classifying protein coding gene (PCG) and long non-coding RNA (lncRNA) genes for their circRNA potential by using features mainly encoded from the primary sequence along with a few other features including conservation. We showed that this yielded performance superior to the basic methods using only features such as GC content and sequence length, demonstrating that this problem is non-trivial. Furthermore, as circular RNAs are getting increasing awareness within the context of stem cells, we included a classifier for circRNAs expressed in stem cells or not. Again, we find that, although less profound, sequence features in general improve the performance over a basic model making use of only GC content and sequence length.

In this study, we used random forest (RF) as the classifier. It has been shown that RF can outperform other methods in many classification tasks [29]. The choice of RF vs. some other machine learning algorithm is not what limits performance. The directions of research that could improve performance is work on obtaining better quality datasets on which to train the models, and possibly more work on feature engineering, i.e., adding new relevant input features for the classifiers.

We expect that the method works, e.g., for other mammals as well based on our mouse case. However, some minor improvements can probably be obtained if training is carried out individual organisms. The models trained on human data were tested on mouse data cleaned for high similarity (80%) to human. WebCricRNA still yields a high accuracy (Figure 4). However, it is still difficult to prove that the method works for other mammals, because, as homology to the human dataset makes it very hard—if possible at all—make a sufficiently large independent datasets for another mammals.

We provide a user friendly web interface by WebCircRNA, which allows the user to either upload a sequence (FASTA format) or a BED file (human genome only), which is a PCG, a lncRNA or a circRNA, to retrieve prediction of the circRNA potential (for PCG and lncRNA) or whether the circRNA is expressed in stem cells. The web server returns and visualizes the score and associated false positive rate from three models, namely CP-PCG, CP-lncRNA and SP-circRNA.

## Figures and Tables

**Table 1:** The details of training and independent test sets. The table summarizes which sequences were used as positive and negative examples for the respective random forest (RF) models.

Model	Positive Data	Negative Data
circRNA vs. PCG	Total: 14,084 circRNAs	Total: 9533 PCGs not overlapping with circRNAs
	Training: 10,000	Training: 8000
	Independent testing: 4084	Independent testing: 1533
circRNA vs. lncRNA	Total: 14,084 circRNAs	Total: 19,722 lncRNAs not overlapping with circRNAs
	Training: 10,000	Training: 10,000
	Independent testing: 4084	Independent testing: 9722
Stem cell vs. not	Total: 2082 circRNAs	Total: 2082 circRNAs
	Training: 1800	Training: 1800
	Independent testing: 282	Independent testing: 282

**Table 2:** The 178 extracted features divided into four groups.

Feature Group	Feature Names
Basic sequence features	Length; AG, GT, GTAG, AGGT, GC content; 64 trinucleotide frequencies
Graph features	Top 101 graph features from GraphProt 1.0.1
Conservation features	Mean, standard deviation of conservation score
Other features	ALU, tandem, ORF length, ORF prop, SNP density

## Abbreviations

The following abbreviations are used in this manuscript:

circRNA	circular RNA
lncRNA	long non-coding RNA
PCG	protein coding gene
ROC	receiver operating characteristic
AUC	the area under the ROC curve
ORF	open reading frame
FPR	false positive rate
CP-lncRNA	circRNA potential of lncRNA
CP-PCG	circRNA potential of PCG
CP-lncRNA	circRNA potential of lncRNA
SP-circRNA	stem cell potential of circRNA
